# Qualité des certificats de coups et blessures volontaires sur adultes à Dakar et Diourbel, Sénégal

**Published:** 2011-12-18

**Authors:** Mohamed Maniboliot Soumah, Hugues Elie Elame Ngwa, Mor Ndiaye, Mamadou Lamine Sow

**Affiliations:** 1 Service de Médecine Légale, Faculté de Médecine, Pharmacie et Odontostomatologie, Université Cheikh Anta Diop (UCAD), Dakar, Sénégal

**Keywords:** Certificat, incapacité, coups et blessures volontaires

## Abstract

**Introduction:**

Le certificat médical est une attestation écrite, destinée à constater un fait d'ordre médical. L'intérêt de cette étude résultait de la fréquence du contentieux entre représentants de la loi et médecins à propos des certificats médicaux descriptifs de coups et blessures volontaires. Les objectifs étaient d’étudier les critères d’établissement des certificats médicaux dans les cas de coups et blessures volontaires, d’évaluer la qualité des certificats médicaux dans les procédures judiciaires dans les régions de Dakar et de Diourbel, d'apprécier l’évaluation de l'Incapacité Totale de Travail fixée par les médecins et leurs implications judiciaires.

**Méthodes:**

Nous avons étudié 201 certificats descriptifs de constatation de coups et blessures volontaires. L'incapacité totale de travail (ITT) au sens pénal a été revue par un médecin légiste. Les données recueillies ont été saisies et analysées par logiciel Epi Data version 3.1 et Epi Info 6.04.

**Résultats:**

Le nom et les prénoms du médecin étaient mentionnés sur les 201 certificats. Sur les 201 certificats étudiés, 170 mentionnaient les faits rapportés par la victime. En proposant de manière comparative pour chacune des ITT fixées par le médecin, une ITT pénale, nous trouvions des erreurs entre ITT pénale et ITT civile (dans 95% des cas).

**Conclusion:**

Les certificats médicaux délivrés sont incomplets. Les recommandations concernent la création d'unités médico-judiciaires et de centres d'accueil des victimes d'agression.

## Introduction

Tout médecin peut être sollicité pour la rédaction d'un “certificat médical” qui contribue à l’établissement de la preuve devant une juridiction. Le certificat médical est une attestation écrite, destinée à constater un fait d'ordre médical. C'est un acte courant de la pratique médicale qui peut être d'ordre pénal et prend une valeur médico-légale, permettant de déterminer indirectement la juridiction compétente et la peine encourue. Il peut être aussi d'ordre civil ou d'ordre social.

L'intérêt de cette étude tient de la fréquence du contentieux entre représentants de la loi (policiers, gendarmes et autres autorités judiciaires) et médecins à propos des certificats médicaux descriptifs de coups et blessures volontaires établis par ces derniers (médecins) et remis aux victimes aux fins de procédures judiciaires. En effet, de plus en plus, ces certificats médicaux sont contestés, sinon purement récusés lors des procédures judiciaires, et il apparait comme une sorte de « course aux certificats médicaux de 21 jours » avec les implications pénales que cela comporte. Cette étude sur les certificats médicaux des coups et blessures volontaires au Sénégal avait pour objectifs d’étudier les critères d’établissement des certificats médicaux délivrés dans les cas de coups et blessures volontaires, d’évaluer la qualité des certificats médicaux dans les procédures judiciaires dans les régions de Dakar et de Diourbel, d'apprécier l’évaluation de l'Incapacité Totale de Travail fixée par les médecins et leurs implications judiciaires; afin de faire des propositions pour une amélioration de la rédaction et de la délivrance des certificats médicaux de coups et blessures volontaires, ainsi qu'une meilleure prise en charge médico-légale des victimes de coups et blessures volontaires.

## Méthodes

Notre étude avait pour cadre géographique les régions de Dakar (capitale du Sénégal) et de Diourbel. La collecte des dossiers était faite au Tribunal Départemental Hors Classe de Dakar et au Tribunal Régional de Diourbel. Nous retenions sur l'ensemble des deux tribunaux, 201 certificats descriptifs de constatation de coups et blessures volontaires (en excluant les violences sexuelles et les violences sur mineurs), certificats rédigés par des médecins de janvier 2006 à juillet 2009. Ces certificats étaient au nombre de 50 pour le Tribunal Départemental Hors Classe de Dakar et de 151 pour le Tribunal Régional de Diourbel. Ils étaient tous inclus dans les dossiers d'affaires déjà jugées, qui comportaient outre ces certificats, d'autres pièces telles que les procès-verbaux de police, les rapports circonstanciés des faits relatés par la victime, l'agresseur et les témoins, les rapports d'enquête, parfois les photographies des lésions, des cartes topographiques de reconstitution des faits... Il s'agissait de certificats rédigés dans le cadre de réquisitions judiciaires ou à la demande des intéressés.

Les certificats inclus dans les dossiers en instance de jugement n’étaient pas inclus à cause du respect du secret de l'instruction. Les paramètres évalués étaient le support (l'entête du papier), la lisibilité, l'identité et la qualité du praticien, la date et l'heure de l'examen, l'identité de la personne examinée (nom et prénoms, date de naissance ou âge, adresse, profession), les commémoratifs, les doléances de la victime, les antécédents médico-chirurgicaux susceptibles d'interférer avec les conséquences des violences subies ou de renseigner sur un état de vulnérabilité, l'examen clinique, les examens complémentaires et traitements, l'incapacité Totale de Travail (ITT au sens pénal) et la signature. L'ITT au sens pénal a été revue par un médecin légiste. Les données recueillies ont été saisies et analysées par logiciel Epi Data version 3.1 et Epi Info 6.04. Les tests statistiques utilisés sont le chi-deux.

## Résultats

### Support

Parmi les 201 certificats étudiés, 186 soit 93% des cas, étaient rédigés sur papier à entête où figuraient le nom de la structure hospitalière et le service. Les 15 autres soit 7% des cas, étaient rédigés sur des papiers ou supports ne donnant aucun renseignement sur le nom de la structure hospitalière.

### Lisibilité

Parmi les 201 certificats, 38 étaient dactylographiés (19%) et 163 étaient écrits à la main (81%). Les certificats dactylographiés ont été tous qualifiés de lisible. Pour les 163 certificats rédigés à la main, 154 d'entre eux étaient parfaitement lisibles et 9 n’étaient pas lisibles.

### Identité du praticien

Pour l'identité du médecin, nous avons tenu compte de trois paramètres: nom et prénoms, qualité du médecin et adresse professionnelle. Le nom et les prénoms du médecin étaient mentionnés sur les 201 certificats. La qualité du médecin était mentionnée sur 164 certificats (82%) et était absente sur 37 certificats (18%). L'adresse professionnelle du médecin était spécifiée sur 180 certificats (90% des cas) et absente sur 21 certificats (10% des cas). Ainsi, l'identité complète du médecin (nom et prénoms + qualité + adresse professionnelle) était retrouvée sur 148 certificats (74%).

### Date et heure de l'examen

La date était notée sur les 201 certificats. L'heure de l'examen n’était précisée qu'une seule fois sur l'ensemble des 201 certificats (0,5%).

### Identité de la victime

Nous considérions quatre paramètres: nom et prénoms, âge ou date de naissance, adresse et profession. Nous recherchions l'emploi du conditionnel par le médecin ou la formule «qui dit se nommer …». Le nom et les prénoms de la victime étaient présents sur tous les certificats de notre série. La date de naissance (ou l’âge) de la victime figurait sur 175 certificats (87%). L'adresse de la victime figurait sur 125 certificats (62%). La profession de la victime était absente sur les 199 certificats (99%).

### Faits rapportés

Nous recherchions les commémoratifs de l'agression narrés au conditionnel, c′est-à-dire: le mécanisme, la date et l'horaire des violences subies. Sur les 201 certificats étudiés, 170 mentionnaient les faits rapportés par la victime (85% des cas).

### Doléances de la victime (plaintes)

Les plaintes de la victime figuraient sur 71 certificats (35%). Parmi les 71 certificats sur lesquels les doléances de la victime étaient rapportées, nous constations que 65 plaintes étaient légitimes, donc en adéquation avec les constatations cliniques du médecin (92%). Sur les 6 autres (8%), les plaintes n’étaient pas en adéquation avec les constatations cliniques du médecin.

### Antécédents

Les antécédents médico-chirurgicaux (susceptibles d'interférer avec les conséquences des violences subies ou de renseigner sur un état de vulnérabilité) figuraient sur 11 certificats (5%).

### Examen Clinique

**Figure 1:**
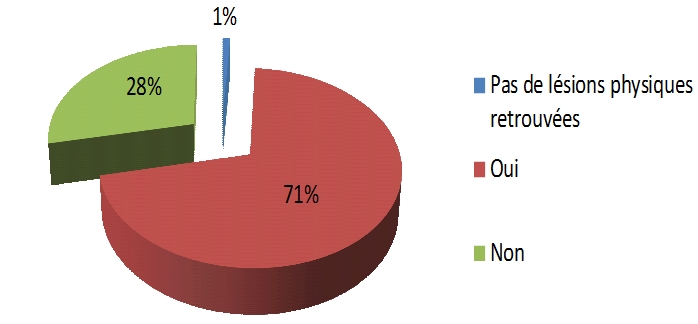
Localisation des lésions par rapport à des repères anatomiques fixes

Parmi les 201 certificats, l'examen clinique était rapporté au présent de l'indicatif sur 191 certificats (95%). Les données anthropométriques et l'examen général étaient rapportés sur 7 certificats (3%). L’évaluation psychique (état émotionnel et état psychique secondaires aux violences subies) était précisée sur 17 certificats (8,5% des cas). La description des blessures physiques présentées par la victime figurait sur 194 certificats (97%). Cinq certificats ne faisaient aucune allusion aux blessures physiques présentées par la victime. Enfin, sur deux certificats, les médecins ont précisé n'avoir retrouvé aucune lésion cliniquement décelable au moment de l'examen. Les blessures retrouvées sont diverses. Par ordre de fréquence décroissante, on notait les plaies (36,4%), les œdèmes traumatiques (17,3%), les fractures (12,6%), les abrasions cutanées (8,9%), les raideurs articulaires (7%), les lésions bucco-dentaires (4,7%), les ecchymoses (4,2%), les hématomes (3,3%), les luxations (2,8%), les brûlures (1,4%), les entorses (0,5%). Sur 177 certificats (88%), des termes précis ont été utilisés pour décrire les blessures physiques de la victime. Sur 22 certificats (11%), on retrouvait une description des blessures physiques de la victime avec des termes imprécis. Sur 142 certificats, les lésions étaient localisées par rapport aux repères anatomiques fixes les plus proches (71%). Sur 57 certificats (28%), la topographie des lésions était précisée, sans donner une localisation fixe par rapport aux repères anatomiques les plus proches (Figure 1). Les lésions se situaient sur le massif crânio-facial (37%), les membres supérieurs et/ou inférieurs (33,5%), le thorax (11%), le dos (7%), l'abdomen/le bassin (6,3%) et le rachis cervical (5,2%).

Les caractéristiques des lésions, c′est-à-dire leurs formes, contours, couleurs et dimensions, étaient précisées sur 72 certificats (36%). Le côté dominant de la victime n’était mentionné sur aucun certificat.

### Examens complémentaires et traitements

Les examens paracliniques et/ou les consultations demandés par le médecin étaient absents sur 166 certificats (83%). Parmi les 201 certificats, 98 précisaient le traitement administré par le médecin (49%).

### Incapacité totale de travail (ITT au sens pénal)

Nous avions retrouvé une ITT attribuée par le médecin sur tous les certificats. Même les deux certificats, sur lesquels les médecins précisaient n'avoir retrouvé aucune lésion cliniquement objectivable, comportaient une ITT.

**Figure 2:**
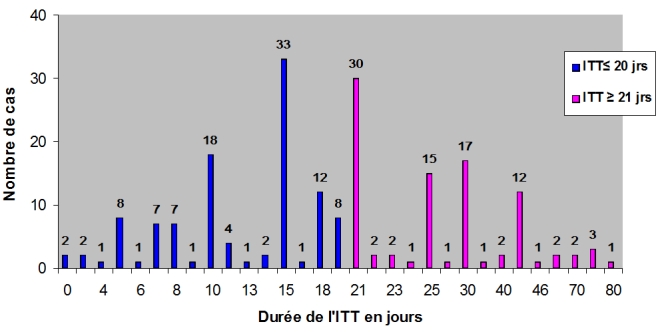
Répartition de la durée d'incapacité totale de travail (ITT) en jours

200 certificats comportaient une ITT exprimée en jours (99,5%) et un seul comportait une ITT exprimée en semaines. Les jours d'ITT attribués variaient entre 0 et 80 jours avec une moyenne de 21,22 jours (Figure 2).

Parmi ces certificats, 108 comportaient une ITT dont la durée était inférieure ou égale à 20 jours (54%). Ainsi, 46% des certificats présentaient une ITT dont la durée dépassait la barre pénale de 20 jours. L'expression « sous réserves de complications » figurait sur 176 certificats (88%).

### Incapacité temporaire totale ou incapacité totale de travail

Nous avons établi une ITT au sens pénal pour 196 certificats. Les valeurs trouvées variaient entre 0 et 30 jours, avec une moyenne de 6,75 jours. Parmi ces 196 certificats, 6 avaient des ITT correspondantes à celles que nous avions proposées (ITT au sens pénal). Il n'y avait convergence entre le médecin traitant et le médecin légiste que sur 6 certificats (3%).

**Figure 3:**
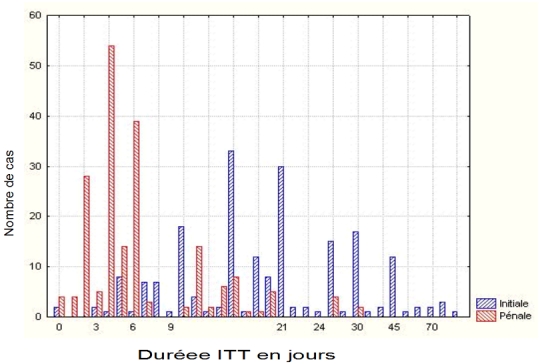
Etude comparée incapacité totale de travail (ITT) fixée et ITT revue en fonction du nombre de cas

Parmi ces 196 certificats dont la durée de l'ITT a été revue, 190 comportaient une ITT dont la durée était inférieure ou égale à 20 jours (97%), et 6 comportaient une ITT dont la durée était supérieure ou égale à 21 jours (3%). En résumé, l'ITT fixée par le médecin correspondait à l'ITT au sens pénal du terme dans seulement 3% des cas (soit 6 cas). Dans 95% des cas (190 cas), l'ITT exprimée par le médecin se rapportait à l'Incapacité Temporaire Totale au sens civil du terme et différait donc de ce fait de l'Incapacité Totale de Travail au sens pénal recherchée en cas de coups et blessures volontaires (Figure 3).

Nous en déduisions les écarts en nombre de jours d'ITT pour 195 certificats, et ces écarts variaient de 0 à 60 jours avec une moyenne de 14,47 jours.

### Signature

Sur tous les certificats figuraient la signature du médecin ayant délivré ledit certificat. Nous avons évalué 201 certificats descriptifs de coups et blessures volontaires sur la base de 23 critères (ou paramètres), et nous avions les résultats suivants: 143 comportaient 7 critères discriminatifs essentiels (71,14%); 188 certificats comportaient au moins 12 critères (93,5%); 13 certificats comportaient au plus 11 critères (6,5%); aucun certificat ne comportait les 23 critères étudiés.

## Discussion

Peu d'enquêtes sénégalaises concernant les coups et blessures volontaires ont été rapportées dans la littérature [1]. Il ressort de notre étude que les certificats médicaux produits par les médecins exerçant dans les régions de Dakar et de Diourbel sont moyennement bien rédigés. Des insuffisances sont à corriger afin d'améliorer la qualité de ces certificats.

Le Louarn [2] dans son étude évaluative sur la rédaction des certificats médicaux en cas de maltraitance à enfant, souligne qu'il est important d'utiliser un papier à entête précisant l'adresse professionnelle du médecin (résidence administrative, centre médico-social…) car il peut exister des situations où les services judiciaires ont besoin de joindre directement le médecin ayant délivré le certificat (pour demande d'informations complémentaires, convocation à une audience), d'où l'importance de la mention de ces coordonnées professionnelles sur ledit certificat.

Un certificat illisible est difficilement exploitable. Hamdouna [3] rappelle que le destinataire final du CMI de coups et blessures volontaires est un non médecin (juriste, autorités judiciaires, agent social ou d'assurance), d'où l'importance de le rédiger de façon claire et lisible, en des termes qui leur sont compréhensibles.

Si le nom du médecin ayant établi le certificat était toujours indiqué dans ce dernier, certains certificats ne mentionnaient pas la qualité du médecin signataire (18% des cas). Ceci rejoint les constatations faites par Le Louarn [2] dans son étude menée à l'Inspection Académique du Bas-Rhin à Strasbourg en France, où il souligne que la mention de la qualité du médecin signataire du certificat donne une valeur probante à cet acte. De plus, nous avons noté que pour ces certificats, en lieu et place de la qualification professionnelle du médecin, figurait le terme « médecin CHU ». Ce terme ne pourrait être considéré comme renseignant sur la qualité du médecin signataire, car il est vague, non spécifique, et ne permet pas de savoir s'il s'agit d'un médecin généraliste, d'un spécialiste, d'un interne des hôpitaux, d'un ancien interne des hôpitaux, d'un étudiant ayant achevé ses études médicales mais non encore titulaire de sa thèse de doctorat, d'un remplaçant non thèsé, d'un médecin installé mais titulaire d'une licence de remplacement, ces derniers [4,5] étant les seuls habilités à délivrer un certificat médical. De ce fait, l'absence de la qualité du médecin signataire pourrait constituer un motif d'invalidité du certificat dans la mesure où toute personne non qualifiée à délivrer un certificat médical peut être punie par la loi d'exercice illégal de la médecine.

Si la date exacte de l'examen figurait sur tous les certificats, l'heure de l'examen n’était signalée que dans 0,5% des cas. A première vue, il peut sembler que la mention de cette dernière soit un fait mineur, mais une fois corrélée à la date et à l'horaire de l'agression (rapportées par la victime), elle trouve tout son intérêt. En effet, certaines lésions comme les ecchymoses ont des caractères colorimétriques qui sont fonction de l’âge de la lésion (durée de la lésion). Ainsi, selon Grill [6], la mention de la date et de l'heure de l'examen, corrélée à la date et à l'heure de l'agression, peut permettre de ressortir la discordance ou la compatibilité entre la date des faits allégués et l'aspect de l'ecchymose; bien plus, en cas d'existence de lésions multiples d’âges différents, la couleur de l'ecchymose (et partant, son âge) est un élément d'orientation. Il est donc souhaitable que ces éléments (dates et heures) soient systématiquement notés sur tous les certificats.

L'entretien avec la victime permet de connaitre son histoire (faits rapportés, date des violences subies…), d'avoir une description des faits à l'origine de ses lésions (mécanismes des violences subies) et ses plaintes (symptômes ressentis). Ce sont des allégations qu'il est préférable de retranscrire au conditionnel ou de mettre entre guillemets en veillant à ne pas les dénaturer [2]. Plus tard, après examen de la victime, le médecin pourra établir selon les constatations qu'il aura faites si les doléances de la victime sont en adéquation avec les lésions retrouvées. Dans notre série, nous constations que 15% de certificats ne mentionnaient pas les plaintes de la victime. Cependant, pour les cas où les plaintes de la victime figuraient sur le certificat, 8% d'entre elles n’étaient pas légitimes. Le fait de relever ces discordances témoigne de l'objectivité du médecin, de son sérieux, mais aussi de sa volonté de rester conforme à l’éthique et à la déontologie médicale.

Nous avions noté une bonne attitude des médecins par rapport à l'indication des examens cliniques. Il est donc important d'attirer l'attention à ce propos car il peut paraître douteux d’établir un certificat médical sans avoir au préalable mentionné un examen physique permettant de renseigner sur la nature des lésions.

La mention des antécédents médico-chirurgicaux susceptibles d'interférer avec les conséquences des violences subies ou de représenter une vulnérabilité médicale est fondamentale. Grill [6] évoque le fait que leur absence peut être préjudiciable à une éventuelle discussion médico-légale notamment en termes d'imputabilité. Elles permettent de plus au magistrat de qualifier d’éventuels facteurs aggravants.

L’état émotionnel et le comportement de la victime observés au cours de l'examen médical ont été peu mentionnés dans les certificats médicaux (8%). Ceci rejoint l’étude menée par Le Louarn et coll. [2] qui soulignent qu'il est recommandé au médecin de décrire les signes psychiques constatés à l'examen médical. Si l'examen médical n'a pas pour objet de poser un diagnostic psychologique, le médecin peut noter le comportement de la victime et/ou ses émotions (évaluation de l’état psychologique) car ce sont des éléments qui vont témoigner de l'existence d'un état de choc secondaire à l'agression (état de choc post traumatique ou état de stress post traumatique). Cet état peut être variable, allant des plus simples manifestations (peur, angoisse…) à des troubles beaucoup plus graves (psychose, dépression…). Signalons par ailleurs que ces troubles du comportement peuvent être tout aussi invalidants pour la victime du point de vue physiologique et fonctionnel que les lésions physiques.

La nature des lésions était variable, avec une prédominance des plaies (36,4% des cas). D'autres études portant sur l’évaluation de la qualité de rédaction de certificats descriptifs de constatations de coups et blessures volontaires menées en France et au Maroc [2,6,7] ont retrouvé la prédominance d'autres lésions. Il s'agit des hématomes et ecchymoses (51%) pour l’étude menée par Benyaich et coll. au sein de l'unité médico-légale du CHU Ibn Rochd de Casablanca [7]. Les plaies occupaient la seconde place (35% des cas). Pour l’étude menée par Grill et coll. au sein de l'unité médico-judiciaire du CHU de Rangueil de Toulouse, les ecchymoses étaient prépondérantes (782 cas), les plaies ne venaient qu'en 4e position (201 cas) respectivement après les dermabrasions et brûlures (759 cas) et les hématomes (295 cas) [6]. Enfin pour ce qui est de l’étude menée par Le Louarn et collaborateurs [2], on notait une prédominance des hématomes et ecchymoses.

Les lésions physiques retrouvées siégeaient préférentiellement au niveau du massif crânio-facial (37% des cas) et des membres supérieurs et/ou inférieurs (33,5% des cas). Les autres localisations étaient moins fréquentes (thorax, dos, abdomen/bassin, et atteintes cervicales). Ceci rejoint l’étude de Benyaich ainsi que celle de Le Louarn, études dans lesquelles les sièges préférentiels des lésions retrouvées étaient également en premier lieu le massif crânio-facial et secondairement les membres. Donc la majorité des lésions physiques observées dans les cas de coups et blessures volontaires portent sur des zones « découvertes » du corps tels que la tête et les membres, visibles par autrui (zone non ou peu cachée par les vêtements). Et à l'inverse, ces lésions sont moins fréquemment retrouvées au niveau des zones « cachées » (tronc, dos…).

Au cours de notre étude, nous avons pu noter 4,7% de lésions bucco-dentaires. Cependant, un fait important est la description approximative de ce type de lésions par les médecins, et ceci s'explique par le fait que ces derniers n'ont pas toujours le temps nécessaire ni la vocation pour un dépistage précis de ce type de lésions. Les lésions dentaires, si elles n'ont jamais de grandes conséquences sur le plan purement médical, peuvent par contre avoir de lourdes répercussions financières pour le patient. C'est notamment le cas lors de la réalisation de prothèses conjointes ou d'implants dans le cadre d'un accident ou d'une agression avec un tiers responsable. Ainsi, il serait donc primordial pour le médecin de signaler sur le certificat initial, même de manière sommaire, l'existence (ou la possibilité) de lésions dentaires, lorsqu'il en constate où en soupçonne l'existence, et dans ce cas-là, il serait également souhaitable d'orienter les patients vers un chirurgien-dentiste ou un stomatologue pour faire établir un certificat complémentaire plus précis sur le plan dentaire.

Les caractéristiques des lésions (formes, dimensions, couleurs, contours) sont peu précisées. Cependant, il faut noter que dans certains cas, des qualificatifs imprécis et non adaptés aux pratiques médico-légales ont été utilisés, et dans d'autres cas, ces caractéristiques ont tout simplement été omises. Ceci rejoint l’étude menée par Grill et coll., qui soulignent le fait qu'un effort de la part des médecins devrait être fait afin qu'il soit systématiquement précisé sur les certificats les caractéristiques des lésions présentées par la victime.

L'impossibilité de localiser certaines lésions de manière satisfaisante dans 28% des cas était liée le plus souvent à une absence de localisation des lésions par rapport à un point fixe. Pour exemple, une dermabrasion arrondie d'un centimètre de diamètre à la face postérieure du tronc sans aucune autre précision est très vague. Elle ne précise pas véritablement à quel niveau se trouve la lésion (partie latérale du dos? partie supérieure? partie inférieure?...). Par contre, dire que l'examen retrouve une érosion arrondie d'un centimètre de diamètre à la face postérieure du tronc, à deux centimètres du bord interne de la scapula (omoplate) gauche est plus précis. Grill souligne le fait qu'il n'existe toutefois pas de techniques systématiques de localisation adaptée à tous les cas. On ne peut qu'insister sur un choix judicieux (qui doit être fait) de la région anatomique de référence. La description d'une lésion par rapport à un point fixe est un élément informatif de certaines discussions médico-légales ou pour certaines localisations lésionnelles pour lesquelles il est difficile de circonscrire une zone anatomique de manière adaptée.

Soulignons l'importance de la mention du côté dominant de la victime qui passe volontiers au second plan, voire carrément inexistante comme c'est le cas dans notre étude où aucun certificat ne précisait le côté dominant de la victime. Et pourtant, Lasseuguette et coll. [8] soulignent son importance dans l’évaluation de la durée de l'ITT personnel. En effet, la gêne fonctionnelle qui découle des lésions présentées par le malade doit être multipliée par deux lorsque ces dernières portent sur le(s) membre(s) du côté dominant. Cette gêne fonctionnelle rentrant elle-même dans les critères de détermination de l'ITT personnel.

La mention des examens paracliniques et/ou des consultations demandées ainsi que celle du traitement administré apparait comme une exigence mineure qui n'influence en rien l'exploitabilité du certificat médical. Aucun des auteurs cités plus haut n'en a accordé une attention particulière. Au cours de ses travaux en vue de la mise sur pied d'un barème indicatif de l'ITT personnel, Lasseuguette fait ressortir l'influence de la prise en charge thérapeutique sur la durée de l'ITT personnelle. En effet le nombre de jours d'ITT personnelle est majoré par la durée d'hospitalisation (si elle existe), il est également majoré par la durée d'immobilisation d'un membre privant le sujet d'une certaine autonomie pour les actes élémentaires de la vie quotidienne. Il faut donc encourager les médecins à davantage préciser sur les certificats les traitements administrés ainsi que les consultations et examens paracliniques auxquels ils ont eu recours.

La mention systématique de la notion d'Incapacité Totale de Travail, en abrégé ITT (était présente sur tous les certificats), souligne le poids important accordé à ce critère souvent considéré comme une des conclusions d'un certificat de constatation de coups et blessures. Cependant, lorsque nous avons eu à proposer de manière comparative pour chacune des ITT fixées par le médecin, une ITT pénale, il y avait des discordances entre ITT pénale et ITT civile (95%). Ce constat est alarmant, du fait que la production d'un certificat médical de coups et blessures volontaires avec une ITT pénale supérieure ou égale à 21 jours implique la garde à vue immédiate de l'auteur des faits avant sa comparution devant le juge correctionnel. Ces écarts au niveau de la durée d'ITT entre celle fixée par le médecin et celle qui aurait dû être normalement attribuée, s'expliquaient par: une non maîtrise de la notion d'Incapacité Totale de Travail personnel ou ITT au sens pénal, ces derniers évaluant le plus souvent la durée de temps nécessaire à la consolidation des lésions, plutôt que d’évaluer le laps de temps durant lequel la victime ne peut accomplir les actes de la vie quotidienne; une fixation sur les certificats médicaux avec une durée d'ITT supérieure ou égale à 21 jours. On pourrait même parler de « course aux certificats de 21 jours ». Cette recherche effrénée est due au fait que les victimes d'agression en plus du dédommagement auquel elles aspirent, veulent voir les auteurs des faits incarcérés. On assiste donc quelque fois à un « véritable marchandage » entre médecins et victimes, pour une surévaluation de la durée d'ITT.

Certains représentants de la loi (policiers, avocats) jouent également un rôle indirect dans cette course effrénée aux certificats de 21 jours, car ils conseillent aux victimes de se procurer un certificat médical attestant d'une Incapacité Totale de Travail personnel d'au moins 21 jours afin que les auteurs des actes soient interpelés.

Le problème relatif à l'ITT n'est pas spécifique à notre étude. Doriat et coll. [9] au décours de leur étude évaluative sur la qualité des certificats médicaux produits par les consultations médico-judiciaires de Lorraine, soulignent le même problème (confusion entre ITT pénale et ITT civile) mais à un degré variable. En effet, si dans notre étude nous avons pu constater 95% d'erreur d'ITT, dans la leur, il n'y avait que 8% de discordances.

Les recommandations concernent le Ministère de la Justice et le Ministère de la Santé, pour la création d'unités médico-judiciaires et de centres d'accueil des victimes d'agression, une collaboration renforcée entre acteurs de la procédure judiciaire et médecins légistes, l’élaboration d'un canevas de formulaire ainsi que d'un barème indicatif de l'ITT pénale, la formation des médecins et des juges à la notion d'ITT au sens pénal du terme.

## Conclusion

Les certificats médicaux descriptifs de coups et blessures volontaires sont moyennement bien rédigés au Sénégal. Ils comportent les critères discriminatifs nécessaires à la validation d'un certificat médical initial descriptif de coups et blessures (71,14% des certificats). Par contre l'incapacité total de travail personnel, elle a été le maillon faible de ces certificats (seulement 3% donnaient une ITT au sens pénal). Un effort doit donc être porté sur cette notion d'ITT qui correspond à la période de perte d'autonomie pour les actes de la vie quotidienne. Son estimation doit tenir compte des lésions objectives, et du retentissement psychique.
